# Surgical preferences of patients at risk of hip fractures: hemiarthroplasty versus total hip arthroplasty

**DOI:** 10.1186/1471-2474-12-289

**Published:** 2011-12-23

**Authors:** Noor Alolabi, Bashar Alolabi, Raman Mundi, Paul J Karanicolas, Jonathan D Adachi, Mohit Bhandari

**Affiliations:** 1 Division of Orthopaedic Surgery, McMaster University, Hamilton, Canada; 2 Department of Surgery, University of Western Ontario, 16-40 Fairfax Crt, London, ON, Canada N6G 3Y3; 3 Memorial Sloan-Kettering Cancer Center, 1275 York Ave., New York, NY, USA 10065; 4 Department of Medicine, McMaster University, Hamilton, Canada

## Abstract

**Background:**

The optimal treatment of displaced femoral neck fractures in patients over 60 years is controversial. While much research has focused on the impact of total hip arthroplasty (THA) and hemiarthroplasty (HA) on surgical outcomes, little is known about patient preferences for either alternative. The purpose of this study was to elicit surgical preferences of patients at risk of sustaining hip fracture using a novel decision board.

**Methods:**

We developed a decision board for the surgical management of displaced femoral neck fractures presenting risks and outcomes of HA and THA. The decision board was presented to 81 elderly patients at risk for developing femoral neck fractures identified from an osteoporosis clinic. The participants were faced with the scenario of sustaining a displaced femoral neck fracture and were asked to state their treatment option preference and rationale for operative procedure.

**Results:**

Eighty-five percent (85%) of participants were between the age of 60 and 80 years; 89% were female; 88% were Caucasian; and 49% had some post-secondary education. Ninety-three percent (93%; 95% confidence interval [CI], 87-99%) of participants chose THA as their preferred operative choice. Participants identified several factors important to their decision, including the perception of greater walking distance (63%), less residual pain (29%), less reoperative risk (28%) and lower mortality risk (20%) with THA. Participants who preferred HA (7%; 95% CI, 1-13%) did so for perceived less invasiveness (50%), lower dislocation risk (33%), lower infection risk (33%), and shorter operative time (17%).

**Conclusion:**

The overwhelming majority of patients preferred THA to HA for the treatment of a displaced femoral neck fracture when confronted with risks and outcomes of both procedures on a decision board.

## Background

Hip fractures are suffered by 280, 000 Americans and 36, 000 Canadians annually [1]. As the population of those aged 65 years or older in North America will rise from 34.8 to 77.2 million by 2040, the incidence of hip fractures is expected to exceed 500, 000 and 88, 000 in the United States and Canada respectively [1]. This increased incidence will correspondingly have an exhaustive economic toll, as health care costs surrounding hip fracture care will reach $9.8 billion in the United States and $650 million in Canada [1,2].

The optimal treatment of femoral neck fractures depends on a multitude of factors including the degree of fracture displacement and patient's age, risk profile, functional demands, cognitive function and physical fitness [3,4]. Three surgical options are available for such fractures: internal fixation, hemiarthroplasty (HA), and total hip arthroplasty (THA) [5,6]. Hemiarthroplasty is the treatment of choice in low-demand and cognitively impaired elderly patients [5]. However, the ideal management of displaced femoral neck fractures in relatively healthy, independent and lucid patients over the age of 60 years remains controversial [5,7]. Most orthopedic surgeons favour arthroplasty over internal fixation [5]. Recent studies have also demonstrated that arthroplasty produces superior functional results relative to internal fixation and is therefore preferable for this patient population [8-10]. However, debate continues among surgeons as to whether HA or THA is optimal, with most surgeons favouring HA [5,11]. Proponents of HA quote the advantages of a shorter and simpler surgical procedure, reduced risk of dislocation and the decreased component costs. On the other hand, advocates of THA criticize HA since it results in rapid wear of the acetabular articular cartilage and emphasize that THA is associated with improved functional outcomes, lower postoperative pain scores and potential decrease in reoperation rates [7,9,10,12-16].

When varying treatment options exist favouring different outcomes and risks, which may be valued differently by patients and physicians, it becomes critical to incorporate and rely on patients' preferences in recommending treatment options [17]. Moreover, information regarding the different surgical options and their outcomes should be delivered to patients in a thorough, consistent and unbiased manner [18]. Decision aids can be invaluable resources to aid surgeons in this communication task. Decision aids are "tools designed to help people participate in decision making about health care options" [19]. They provide clear information on the treatment options as well as their risks and benefits and thus assist patients clarify and communicate the personal value they associate with different features of the options [19].

Despite the controversy surrounding hip fracture care, the orthopaedic literature is void of studies eliciting patient's preferences and detailing the effectiveness of decision aids to inform patients of hip fracture management [20]. The purpose of this study was to utilize a decision board to elicit surgical preferences for treatment of displaced femoral neck fractures from patients at risk for sustaining this fracture.

## Methods

### Decision aid development

We developed an electronic decision board following accepted decision board development methods [21-25]. The decision tool fulfilled the CREDIBLE criteria for assessing decision tool quality [26].

The contents of the decision board were abstracted from a systematic review conducted to identify all randomized controlled trials comparing HA and THA for the treatment of displaced femoral neck fractures in patients over the age of 60 years. We searched articles using MEDLINE and EMBASE from 1966 to January 2007. No language restrictions were applied. The bibliographies of all retrieved publications were reviewed for additional relevant articles. The article titles and abstracts were then assessed to ensure that the study was a randomized trial involving the treatment of displaced femoral neck fractures with HA or THA. The data abstracted from the eligible studies consisted of interventions, duration of surgery, functional outcomes and rates of complications (infections, dislocations, medical complications, reoperations and mortality).

Five randomized controlled trials met the inclusion criteria [9,10,16,27,28]. The paper by Ravikumar [9] represents a 13-year follow-up of the Skinner trial [10] and therefore it was not used for our data abstraction. The results of the remaining 4 studies were pooled across studies using estimates from individual trials weighted based on sample size to produce the data presented in the electronic decision aid (Table 1).

**Table 1:** Derivation of the pooled data used in the decision board based on the outcomes from the included clinical trials

Outcomes	THA	HA
**Operative Time**		

Keating	80	59

Dorr	-	-

Baker	93	78

Skinner	-	-

*Pooled Data*	*87 min*	*69 min*

**Post-operative Walking Distance**		

Keating	-	-

Dorr	-	-

Baker	3.6 Km	1.9 Km

Skinner	-	-

*Pooled Data*	*3.6 Km*	*1.9 Km*

**Residual Pain**		

Keating	29/61	30/60

Dorr	-	-

Baker	-	-

Skinner	0/62	20/73

*Pooled Data*	*29/123 (24%)*	*50/133 (38%)*

**Failure to Regain Mobility**		

Keating	-	-

Dorr	7/39	15/50

Baker	-	-

Skinner	13/62	11/73

*Pooled Data*	*20/101 (20%)*	*26/123 (21%)*

**Dislocation**		

Keating	3/69	2/69

Dorr	7/39	2/50

Baker	3/40	0/41

Skinner	10/80	11/100

*Pooled Data*	*23/228 (10%)*	*15/260 (6%)*

**Medical Complications**		

Keating	14/69	12/69

Dorr	-	-

Baker	1/40	3/41

Skinner	-	-

*Pooled Data*	*15/109 (14%)*	*15/110 (14%)*

**Superficial Wound Infection**		

Keating	2/69	2/69

Dorr	0/39	0/50

Baker	2/40	1/41

Skinner	-	-

*Pooled Data*	*4/148 (3%)*	*3/160 (2%)*

**Deep Wound Infection**		

Keating	1/69	1/69

Dorr	0/39	0/50

Baker	1/40	0/41

Skinner	1/80	0/100

*Pooled Data*	*3/228 (1%)*	*1/260 (0%)*

**Reoperation within 1 year**		

Keating	6/69	5/69

Dorr	9/39	6/50

Baker	1/40	6/41

Skinner	13/80	24/100

*Pooled Data*	*29/228 (13%)*	*41/260 (16%)*

**3-4 month Mortality**		

Keating	2/69	5/69

Dorr	-	-

Baker	-	-

Skinner	8/80	15/100

*Pooled Data*	*10/149 (7%)*	*20/169 (12%)*

**1 year Mortality**		

Keating	4	6

Dorr	-	-

Baker	-	-

Skinner	18	27

*Pooled Data*	*22/149 (15%)*	*33/169 (20%)*

**Figure 1:**
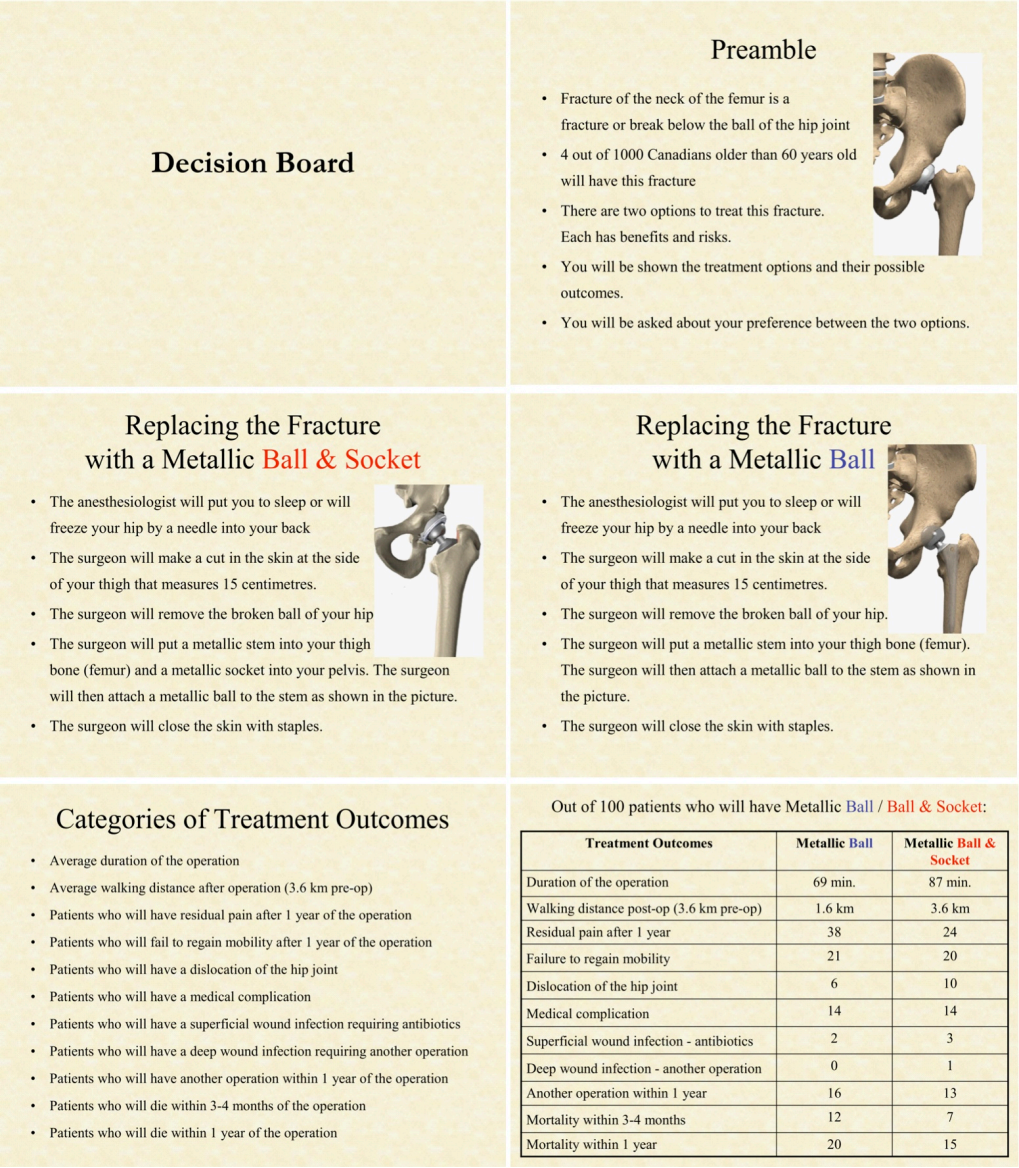
**An Illustration of the decision board**.

The information included in the electronic decision board (Figure 1) was based on a previously developed orthopedic decision board addressing treatment options for displaced femoral neck fractures [29]. This information included a background about femoral neck fractures, a description of the two treatment options [30] (HA and THA) and their respective outcomes and risks. We used the terms "metallic ball" and "metallic ball and socket" to avoid bias by introducing the words "hemi/partial" and "total". We presented the outcomes as probabilities using the phrase, "Out of 100 patients who will have this procedure, a certain number will develop the complication indicated."

Decision boards have been extensively tested in multiple medical fields including orthopedic surgery, and shown to be valid, reliable and easily administered tools [21,31,32]. We were confident in the reliability of this decision board given our previous evaluation of a similar decision board on femoral neck fractures [29].

### Participants

Approval was obtained from the local research ethics board for the study. Participants were recruited from an osteoporosis clinic. Participants were all independent, competent and over the age of 60 years. This population was selected as the study subjects because older patients with osteoporosis would represent a high-risk group to suffer femoral neck fractures [14,33-38].

Participants were excluded if they were incompetent cognitively or if they had a previous hip fracture or replacement, since their choice could be biased towards or against the treatment option they previously received. Patients arriving at the osteoporosis clinic were screened for eligibility; if they met the criteria, then they were asked to participate and included in the study if they provided consent.

### Decision board administration

The decision board was administered to participants by two of the authors (N.A. and R.M.). After the board was developed, a guideline outlining what and how information would be presented was established between the two interviewers. In addition, the interviewers administered the decision board to each other to ensure consistency in the way the interview was conducted.

Participants were presented with the following hypothetical scenario: "While walking, you slip on ice and fall down. You are taken to the nearest hospital and after careful physical and radiographic examination, you are found to have a femoral neck fracture (a fracture of the long thigh bone at the hip level). The orthopedic surgeon tells you that there are two treatment options for this type of fracture." The decision board was then presented to the participants, who read each part of the decision board independently. Questions were encouraged at any time during the interview to clarify vague information or address any concerns. All questions were answered ensuring that the interviewers elicited no bias. Participants' stated their preferences for treatment and the strength of their choice using a 7-point adjectival scale (1--I definitely prefer metallic ball and socket; 4--I am indifferent; 7--I definitely prefer metallic ball). They were then asked for the primary reasons of their treatment option choice.

### Demographic and satisfaction questionnaire

At the end of the interview, the participants completed a questionnaire on socio-demographic variables (gender, age, race, educational level, occupation, and income), previous history of fractures, and an evaluation for the acceptability and satisfaction of using the decision board as a tool to inform patients about treatment options. Acceptability of the decision board was assessed by asking questions regarding how well they understood the board, its usefulness in helping them make a decision, and whether they would recommend it for others. Satisfaction was assessed with respect to the amount of information provided, the use of the decision board as a method to present material and its use of the as a decision-making tool.

### Data analysis

For each study, we calculated the means differences for continuous outcomes. When possible, we pooled the estimates from individual trials based on sample size. Group data were summarized in terms of frequency and percentage (with 95% confidence intervals). With approximately 70% of participants choosing one surgical option, we determined that a sample size of 80 would be needed for a 95% confidence interval +/-10%.

## Results

Between May 2008 and May 2009, we screened participants in the osteoporosis clinic for eligibility. Eight-four participants met eligibility criteria and were recruited. Three participants were omitted from the study post-interview; one had Alzheimer's disease and two did not understand English well. Data was therefore collected and analyzed for 81 participants.

Eighty-five percent (85%) of participants were between the age of 60 and 80 years; 89% were female; 88% were Caucasian; and 49% had some post-secondary education (Table 2). By far, the majority of participants had a documented diagnosis of osteoporosis. The remainder minority had predisposing factors putting them at a high risk for developing osteoporosis.

**Table 2:** Participants' Demographics

Demographic Category		THA (n = 75)	HA (n = 6)	Total (n = 81)
**Gender**	Male	6 (8%)	3 (50%)	9 (11%)
	
	Female	69 (92%)	3 (50%)	72 (89%)

**Age**	60-70 y.o	38 (51%)	1 (17%)	39 (48%)
	
	70-80 y.o	27 (36%)	3 (50%)	30 (37%)
	
	80-90 y.o	9 (12%)	2 (33%)	11 (14%)
	
	> 90 y.o	1 (1%)	0 (0%)	1 (1%)

**Race**	White or Caucasian	67 (89%)	4 (67%)	71 (88%)
	
	Asian	1 (1%)	0 (0%)	1 (1%)
	
	Black	2 (3%)	0 (0%)	2 (2%)
	
	South Asian	2 (3%)	1 (17%)	3 (4%)
	
	Other	3 (4%)	1 (17%)	4 (5%)

**Education Level**	High School	28 (37%)	2 (33%)	30 (37%)
	
	Post-Secondary	38 (51%)	2 (33%)	40 (49%)
	
	Other	9 (12%)	2 (33%)	11 (14%)

**Income **(n = 55 for THA, 5 for HA and 60 for Total)	$0-$40, 000	31 (56%)	5 (100%)	36 (60%)
	
	$40, 000-$80, 000	24 (44%)	0 (0%)	24 (40%)
	
	> $80, 000	0 (0%)	0 (0%)	0 (0%)

**Figure 2:**
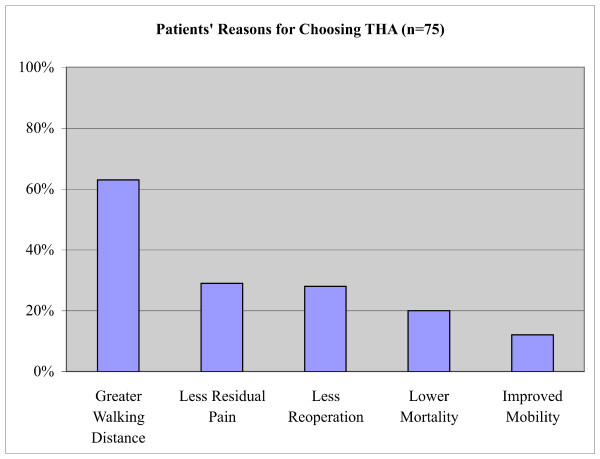
**Patients' Cited Reasons for Preferring THA**.

**Figure 3:**
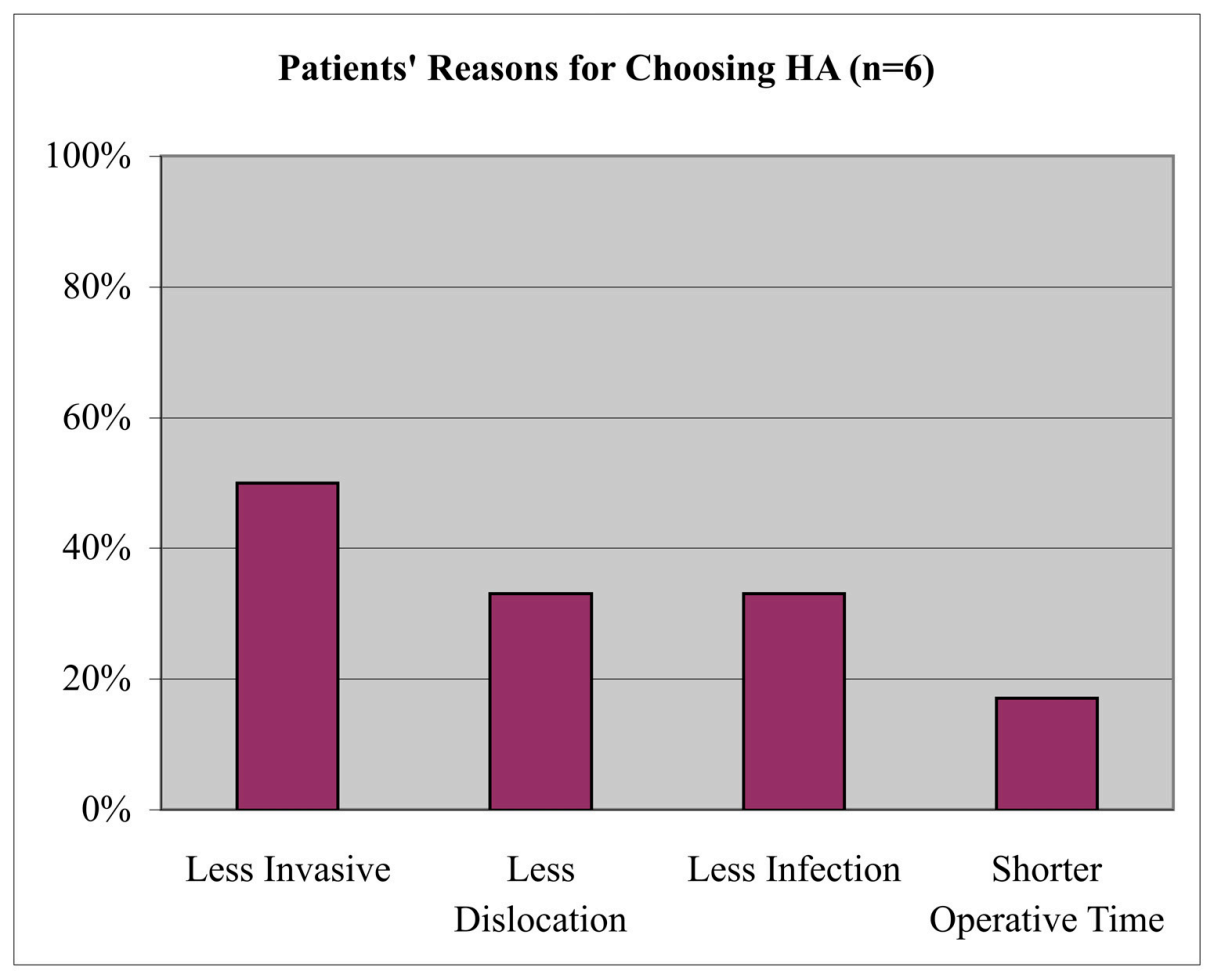
**Patients' Cited Reasons for Preferring HA**.

**Figure 4:**
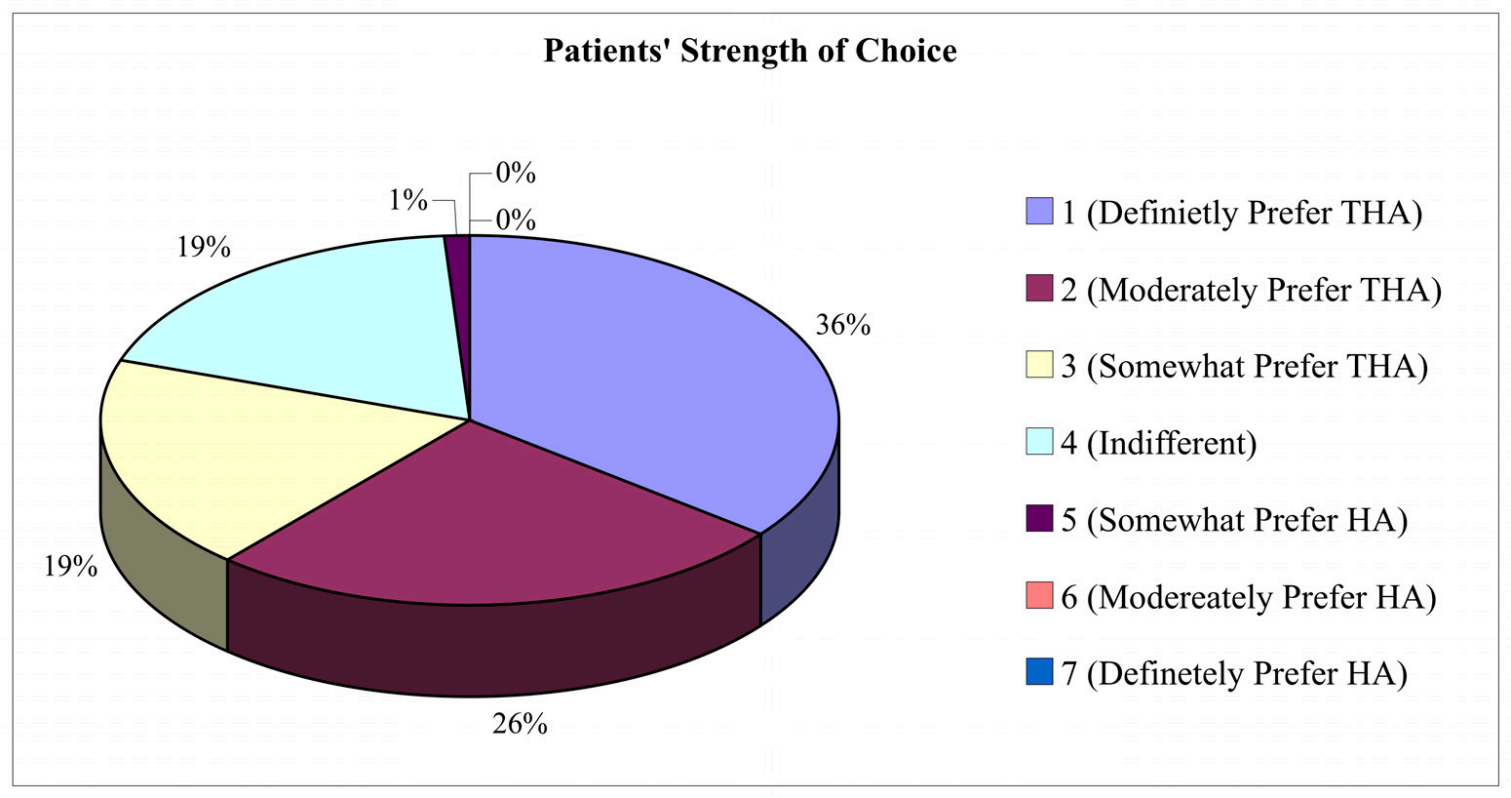
**Patients' Strength of Choice**.

Of 81 participants, 75 (93%; 95% confidence intervals [CI], 87-99) chose THA as their preferred treatment option. The main factors contributing to this choice included the perception of ability to walk a greater distance (63%; CI, 52-74), less residual pain (29%; CI, 19-39), lower reoperative risk (28%; CI, 18-28), and lower mortality (20%; CI, 11-29) (Figure 2). Participants who chose HA (7%; CI, 1-13) as their preferred treatment choice cited less invasiveness (50%; CI, 39-61), less dislocation (33%; CI, 23-43), less infection (33%; CI, 23-43), and shorter operative time (17%; CI, 9-25) (Figure 3). The majority of those who chose THA were quite comfortable with their preference as indicated by their strength of choice, whereas the majority of those who chose HA were closer to being indifferent (Figure 4).

**Figure 5:**
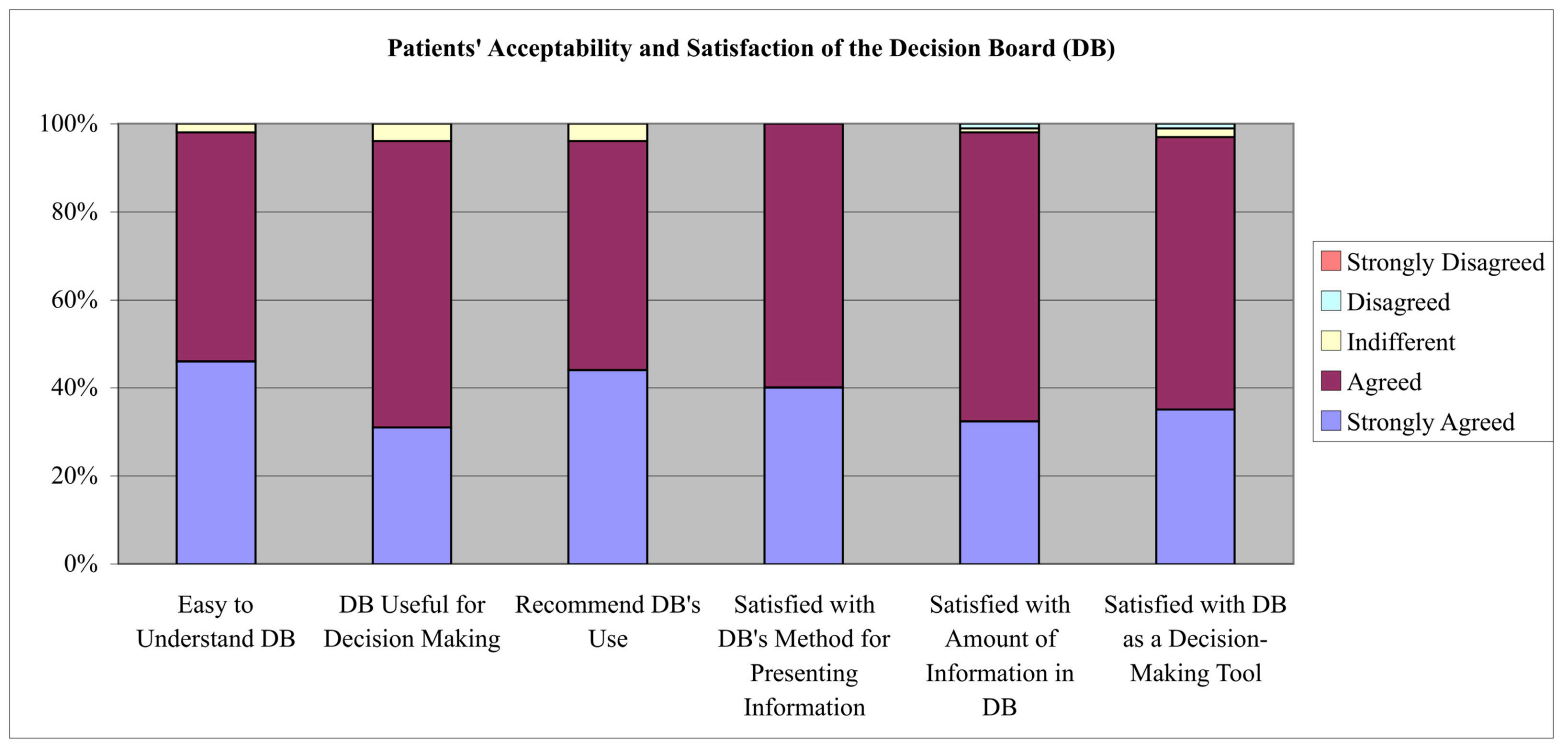
**Patients' Acceptability and Satisfaction with the Decision Board**.

All participants completed the acceptability and satisfaction questionnaire. Ninety-eight percent (98%; CI, 95-101) of participants stated that the decision board was easy to understand, 96% (CI, 92-100) reported that it helped them make a decision, and 96% (CI, 92-100) indicated that they would recommend the use of the decision board to others. Furthermore, 100%, 97% (CI, 93-101) and 97% (CI, 93-101) of participants were satisfied with the decision board as a method for presenting information, the amount of information presented, and the use of the decision board as a decision-making tool respectively (Figure 5).

## Discussion

This decision board analysis demonstrates that the overwhelming majority of participants preferred total hip arthroplasty to hemiarthroplasty for the treatment of displaced femoral neck fractures in patients over 60 years. The main contributing factors for this preference were greater post-operative walking distance, less residual pain, and reduced risk of reoperation. Interestingly, most orthopedic surgeons have the opposite preference; an international survey by Bhandari et al. as well as a survey administered to the American Association of Hip and Knee Surgeons both illustrated that 80-85% of surgeons favor hemiarthroplasty over total hip arthroplasty for the treatment of displaced femoral neck fractures in patients over 60 years [5,11].

Our findings demonstrate a substantial discrepancy between patient and surgeon preferences for the treatment of displaced femoral neck fractures. Other studies have demonstrated that there is a gap between patient and physician preferences [39,40]. For example, compared to physicians, patients are willing to accept a much higher bleeding risk for an associated reduction in risk of stroke [39], and cancer patients are willing to accept treatment with severe side effects for a much smaller chance of improvement [40]. It is therefore important for clinicians not to assume their patients' preference and to ensure that their patients are well informed about treatment options in an unbiased manner.

Involving patients in the decision-making process is the focus of patient-centered care. Studies have revealed that involving patients in their care changes patient behavior and increases compliance with treatment [41,42]. Decision aids can be invaluable resources, assisting physicians to relay unbiased information to patients during this shared decision-making process [18,20]. A systematic review of 55 randomized controlled trials evaluating the efficacy of decision aids compared to no aid, usual care, or an alternative intervention, illustrated that patients who used a decision aid were more satisfied and had greater knowledge and less decisional conflict relative to patients in the other groups [26]. In our study, the majority of patients found the decision board easy to understand, enabled them to make a decision and were satisfied with it as a method to present information and as a decision making tool.

At the time of the onset of our study, only the trials included in our study were published. However, there have been a number of trials and reviews published since then, most favoring THA over HA [8,12-14,43,44] and others showing no advantage of THA over HA and thus favoring HA [45]. Although we acknowledge that these new studies may affect the results of our study, however, most studies still demonstrate similar trends to our decision board. Since the controversy regarding THA and HA for the treatment of displaced femoral neck fractures still exists, eliciting patients' preferences is important.

The treatment choices presented to the participants in our study included HA and THA. A non-operative approach was not included since it is restricted to a very narrow patient group with significant medical conditions preventing them from operative treatment. Similarly, internal fixation was not considered because we have previously performed a similar study eliciting treatment preferences between internal fixation and hemiarthroplasty [29].

Our study has several strengths. We followed rigorous, well-defined methodology to develop the decision aid. We used one-on-one interviews to ensure that all participants understood the questions being asked and provided their true preferences. All participants were above 60 years and 85% of them were between the ages of 60 and 80--the main controversial age group for treatment of these fractures. Furthermore, the patients were recruited from an osteoporosis clinic and had no previous history of hip fracture, which made them a high-risk population for intracapsular hip fractures yet avoided biases such as chronic pain secondary to osteoarthritis or a prior hip replacement with HA or THA.

The main limitation of this study is the study population. Ideally we would have involved real patients with displaced intracapsular hip fractures at the point of decision-making. However, this task is a challenging one since most surgeons often have strong preferences about the appropriate treatment modality of individual patients [13]. Also, at our institution, and many other institutions, THA is performed only by specially trained arthroplasty surgeons, who represent a small portion of surgeons treating acute femoral neck fractures. Hence, we could not administer the decision board and elicit preferences in actual hip fracture patients since it would not be possible or practical to offer all patients THA if they chose it as their preferred treatment option. However, the study population we studied was perhaps the closest to real patients. The typical patient presenting with a femoral neck fracture is a woman over 60 years with osteoporosis and other comorbidities [14,33-38]. Osteoporosis and prior fragility fractures are the most significant risk factors for hip fracture. Many other risk factors including age, female sex, family history of fractures and body mass are indirect measures or risk factors of osteoporosis [34]. We chose independent and competent patients over the age of 60 years who were at an osteoporosis clinic either for treatment of a documented diagnosis of osteoporosis or a condition predisposing them to develop osteoporosis. Also, most of our patients were females. Therefore, we believe that this patient population is highly representative of actual independent and relatively active patients with an intracapsular hip fracture. Another limitation is the fact that newer trials comparing THA and HA are not included in the decision board data but, as mentioned, these studies were not published at the time of onset of this study.

## Conclusion

The overwhelming majority of patients prefer THA to HA for the treatment of displaced femoral neck fractures, despite the current trend of surgeons to favor HA over THA. Surgeons should discuss the advantages and disadvantages of each approach with individual patients and involve them in a shared decision-making process. Decision aids may be helpful to surgeons in this process, as well as other areas of surgical management.

## Competing interests

The authors declare that they have no competing interests.

## Authors' contributions

NA interviewed the majority of the patients, acquired the data and contributed in writing the manuscript. BA designed the study and decision board, obtained REB approval and assisted with data analysis and manuscript preparation. RM interviewed the remainder of the patients, acquired the data and contributed in writing the manuscript. PJK was involved in the study design, assisted with data analysis and helped with manuscript preparation. JDA was involved in patient recruitment and interviews and assisted with manuscript preparation. MB is the study supervisor; he assisted with study design and manuscript preparation. All authors read and approved the final manuscript.

## Pre-publication history

The pre-publication history for this paper can be accessed here:

http://www.biomedcentral.com/1471-2474/12/289/prepub
